# Evaluation of the combined efficacy of carvone and colistin against colistin-resistant *Pseudomonas aeruginosa*: *in vitro* and *in vivo* studies

**DOI:** 10.1128/spectrum.02307-25

**Published:** 2026-05-14

**Authors:** Zeyong Zhong, Zhexiao Ma, Yichi Zhang, Yanchun Gong, Yunying Ge, Yuhan Yang, Tieli Zhou, Jianming Cao

**Affiliations:** 1 School of Laboratory Medicine and Life Science, Wenzhou Medical University 26453, Wenzhou, Zhejiang, China; 2 Department of Clinical Laboratory, The First Affiliated Hospital of Wenzhou Medical University; Key Laboratory of Clinical Laboratory Diagnosis and Translational Research of Zhejiang Province 89657, Wenzhou, Zhejiang, China; Oklahoma State University, Stillwater, Oklahoma, USA

**Keywords:** colistin, carvone, *Pseudomonas aeruginosa*, antibiofilm, synergistic effect

## Abstract

*Pseudomonas aeruginosa* (*P. aeruginosa*) is the primary pathogenic bacterium causing nosocomial-acquired infections, particularly in immunocompromised individuals. Owing to the abuse of colistin (COL), a frontline drug in clinical settings, the resistance rate of *P. aeruginosa* to COL has been escalating year by year, exerting a severe impact on public health. To solve this clinical problem, new therapeutic approaches are urgently required. This study initially proposes a strategy of using carvone in combination with COL to combat colistin-resistant *Pseudomonas aeruginosa* (COL-R *P. aeruginosa*). The checkerboard assay and time-kill assay validated the *in vitro* antibacterial action of this combination against COL-R *P. aeruginosa*. Furthermore, crystalline violet staining and scanning electron microscopy (SEM) confirmed that the combined treatment effectively inhibited biofilm formation. Moreover, the thigh infection model in neutropenic mice showcased the *in vivo* effectiveness of the combined therapy. In addition, the erythrocyte hemolysis experiment and the *in vivo* safety assessment revealed that the combination of carvone and COL exhibited a certain level of safety at effective concentrations. The synergistic antibacterial and antibiofilm mechanisms of carvone combined with COL may be associated with the inhibition of quorum-sensing gene expression, enhancement of bacterial inner and outer membrane permeability, and increased generation of reactive oxygen species. This study offered a new therapeutic strategy for COL-R *P. aeruginosa* infections.

**IMPORTANCE:**

The increasing prevalence of colistin-resistant *Pseudomonas aeruginosa* (COL-R *P. aeruginosa*) poses a significant threat to global public health, as it limits treatment options for severe infections. This study investigates the synergistic effects of carvone, a natural compound, combined with colistin (COL) to combat COL-R *P. aeruginosa* both in *vitro* and *in vivo*. The findings reveal that carvone can restore the susceptibility of COL-R *P. aeruginosa* to COL. By exploring this novel combination, the research aims to provide a potential alternative therapeutic strategy for COL-R *P. aeruginosa* infections. The findings could pave the way for new approaches to treat infections caused by this resilient pathogen.

## INTRODUCTION

*Pseudomonas aeruginosa* (*P. aeruginosa*) is an opportunistic pathogen causing nosocomial infections and ranks fourth among major nosocomial pathogens after *Escherichia coli*, *Klebsiella pneumoniae*, and *Staphylococcus aureus*. Due to its rapid mutation and biofilm formation, *P. aeruginosa* often exhibits multi-drug resistance (MDR), making clinical treatment extremely challenging. It has been placed in the “critical” category on the World Health Organization’s (WHO) list of bacterial pathogen priorities, highlighting the urgent need for the development of new treatment strategies (1–3). Colistin (COL), an older lipopeptide antibiotic, saw reduced clinical use by the mid-1970s due to nephrotoxicity and neurotoxicity (4). However, the rise of MDR Gram-negative bacteria (GNB) in the mid-1990s led to COL’s resurgence as a last-resort treatment. Excessive and improper use has now resulted in global COL resistance, including in *P. aeruginosa* (COL-R *P. aeruginosa*) (5). This poses a significant clinical challenge, necessitating innovative approaches like new drug combinations (6). Combination therapy can overcome COL resistance, reduce its effective concentration, and minimize toxicity, making it an effective strategy (7).

COL mainly targets the outer membrane of GNB, especially the lipopolysaccharide (LPS) layer (8). Contact and interaction with lipid A on LPS, it leads to enhanced and damaged permeability of the bacterial membrane and ultimately lyses the bacteria (9). Most COL-R strains modify LPS through various mechanisms, causing COL to lose its target site and thereby generating resistance to COL (10). The common mechanisms of COL-R are mediated by chromosomes, with the two-component systems PmrA-PmrB (PmrAB) and PhoP-PhoQ (PhoPQ) contributing significantly to COL-R (6). The PmrB and PhoQ proteins possess tyrosine kinase activity that phosphorylates the regulatory proteins (PhoP or PmrA), thereby activating the *pmrHFIJKLM* operon, which catalyzes the addition of positively charged moieties (4-amino-4-deoxy-L-arabinose or phosphoethanolamine) to lipid A. This ultimately reduced the adsorption of COL (positively charged), thereby leading to COL-R (10). Furthermore, the formation of bacterial biofilms is also a significant mechanism mediating antibiotic resistance. Biofilms can not only act as a barrier to assist bacteria in resisting the action of antibiotics, but also, as indicated by some studies, promote the horizontal transfer of drug resistance genes within them (11). Among them, *P. aeruginosa* possesses a powerful ability to form biofilms; thus, it is of paramount importance to inhibit the formation of its biofilms (12).

Carvone is a ketone monoterpene primarily found in essential oils of plants from the Mentha genus. It comes in the (R)-(−) and (S)-(+) isomeric forms and is extensively utilized in the food, pharmaceutical, cosmetic, and agricultural sectors (13–15). Its safety has been recognized by regulatory authorities in multiple countries and regions, and studies have demonstrated that carvone exhibits significant therapeutic potential in areas such as anti-infection, anti-tumor, anti-inflammatory, antispasmodic, neuroprotection, and metabolic regulation (13). These isomers have identical chemical and physical properties, but studies have demonstrated that (S)-(+)-carvone exhibits stronger antimicrobial activity against Gram-negative bacteria (16). Studies have shown that carvone may serve as an adjunct antibacterial agent against local *P. aeruginosa* infections, and carvone is relatively safe without genotoxicity at conventional doses (13, 17). Currently, there are no reports on the combination of carvone and COL. However, there is clear and well-documented evidence that carvone acts as an enhancer of antibiotic activity (17, 18).

In this research, we employed (S)-(+)-carvone as an adjunct antibacterial agent in combination with COL to confront COL-R *P. aeruginosa*. The antibacterial activity of this combined treatment was investigated both *in vitro* and *in vivo*, and the inhibitory effect on the biofilm formation of *P. aeruginosa* was evaluated. This provides a new therapeutic approach for directing the clinical management of *P. aeruginosa* infections.

## RESULTS

### Antimicrobial resistance profiles and background information of strains

We chose 12 unique clinical *P. aeruginosa* isolates for the experiment. Table S1 showed that all strains of *P. aeruginosa* were resistant to COL, with the minimum inhibitory concentrations (MICs) ranging from 8 to 32 µg/mL. The MICs of carvone for the test strains were all greater than 512 µg/mL. Seven of these strains exhibited resistance to at least three antibiotics, indicating that they were MDR. Table S1 demonstrates that these 12 strains are classified into 10 distinct sequence types, which preliminarily indicates that the strain collection in this study exhibits good genetic diversity and is not derived from the spread of a few dominant clones. This strongly supports the representativeness of the subsequent experimental results of this study for the clinical COL-R *P. aeruginosa*. Table S2 presents the COL-R mechanisms of these experimental strains, which involve mutations related to the PmrAB or PhoPQ systems.

### Checkerboard assay shows synergistic effect

Table 1 shows that the combination of carvone and COL exhibited a significant synergistic effect (fractional inhibitory concentration index [FICI] ≤ 0.5) against all COL-R *P. aeruginosa* strains. More intriguingly, carvone could reduce the MIC of colistin for these strains to 1 µg/mL or lower. Furthermore, based on the COL susceptibility breakpoint (2 µg/mL) set by the Clinical and Laboratory Standards Institute (CLSI), it can be concluded that carvone reinstated the susceptibility of these COL-R *P. aeruginosa* strains to COL. Table S3 shows that carvone synergizes with COL in clinically frequent COL-R *Escherichia coli* and *Klebsiella pneumoniae*; however, it does not lower the MIC below the susceptibility breakpoint. Furthermore, carvone does not exhibit a synergistic effect when combined with other antibiotics commonly used in the clinical treatment of *P. aeruginosa* (ciprofloxacin and tobramycin) (Table S4). Therefore, this study primarily focuses on the combination of carvone and COL against COL-R *P. aeruginosa*.

**TABLE 1:** FICI values for colistin/carvone combinations against colistin-resistant *P. aeruginosa^a^^,^^b^^,^^c^*

Strains	Monotherapy MIC (μg/mL)		Combination MIC (μg/mL)		FICI	Interpretation
	Carvone	COL	Carvone	COL		
TL7733	>512	8	32	0.5	<0.125	Synergistic
TL1671	>512	32	128	0.5	<0.266	Synergistic
TL2917	>512	8	64	0.25	<0.156	Synergistic
TL7929	>512	32	64	0.5	<0.141	Synergistic
TL2314	>512	8	128	0.5	<0.313	Synergistic
TL8126	>512	32	128	0.5	<0.266	Synergistic
TL7333	>512	16	64	0.25	<0.141	Synergistic
TL7508	>512	8	32	0.25	<0.094	Synergistic
TL7505	>512	8	16	1	<0.156	Synergistic
TL7548	>512	16	128	0.5	<0.281	Synergistic
TL7440	>512	16	64	1	<0.188	Synergistic
TL8269	>512	8	32	0.5	<0.125	Synergistic

^*a*^ The fractional inhibitory concentration index (FICI) was computed to assess the synergistic effect of the combination of two drugs.

^*b*^ FICI = FIC_Carvone_ + FIC_COL_ = (MIC_Carvone in combination_/MIC_Carvone alone_) + (MIC_COL in combination_/MIC_COL alone_).

^*c*^ FICI ≤ 0.5 indicates a synergistic effect, 0.5 ˂ FICI ≤ 4 indicates no interaction, and FICI > 4 indicates an antagonistic effect.

### Time-kill assay further demonstrates synergistic effect

**Fig 1:**
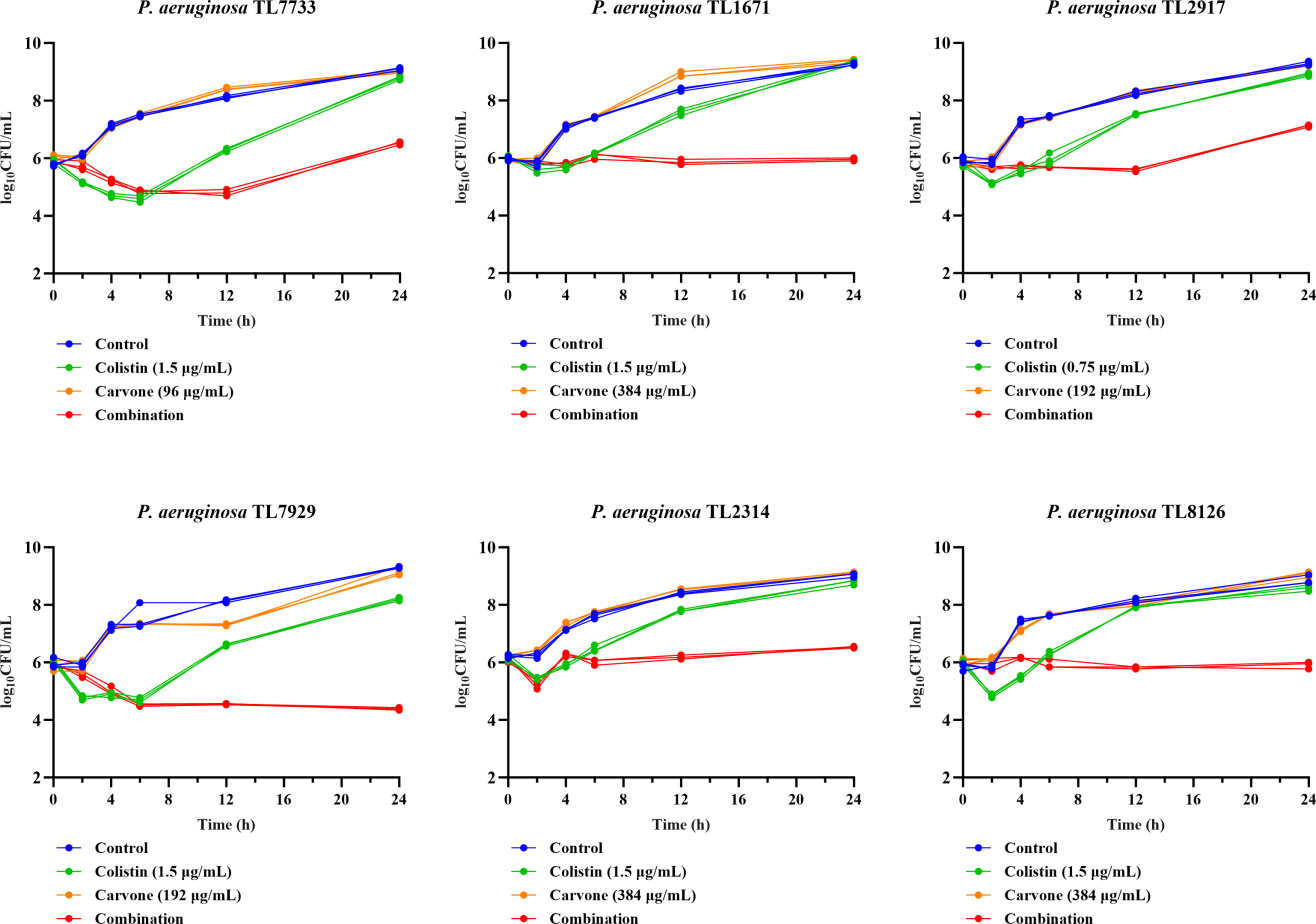
Time-kill assay of carvone and COL individually or in combination against COL-R *P. aeruginosa*.

The impact of carvone and COL on the growth kinetics of COL-R *P. aeruginosa* was investigated using a time-killing assay. The selection of drug concentrations (the concentrations of COL and carvone were 3 × MIC_COL in combination_ and 3 × MIC_Carvone in combination_, respectively) considered the results of the checkerboard assay and the dynamic culture conditions employed in this experiment. Figure 1 shows that the combined treatment group initially had a potent inhibitory effect on test strains similar to COL monotherapy for the first 6 h. However, COL monotherapy ceased to inhibit strain growth beyond this period, whereas the combined treatment continued inhibiting until 12 h. However, with further extension of time, regrowth of certain strains (TL7733 and TL2917) was observed in the combined treatment group, although the bacterial counts remained significantly lower than those in other groups. This indicates that the combined treatment group exhibited significant antibacterial effects (compared to the control group, the bacterial load was reduced by at least 2 log_10_ CFU/mL within 24 h), but the efficacy declined after 12 h (some strains undergo regrowth). The time-kill curves of carvone monotherapy were nearly identical to those of the control group, whereas the curve for COL monotherapy exhibited a change in slope after 6 h, transitioning to an upward trend. Ultimately, at the 12 to 24 h time points, the bacterial load in the COL monotherapy group indeed rebounded to levels comparable to those of the control group. This indicates that monotherapy was ineffective in suppressing the growth of the strain.

### Synergistic effect *in vivo* antibacterial activity of carvone and COL

**Fig 2:**
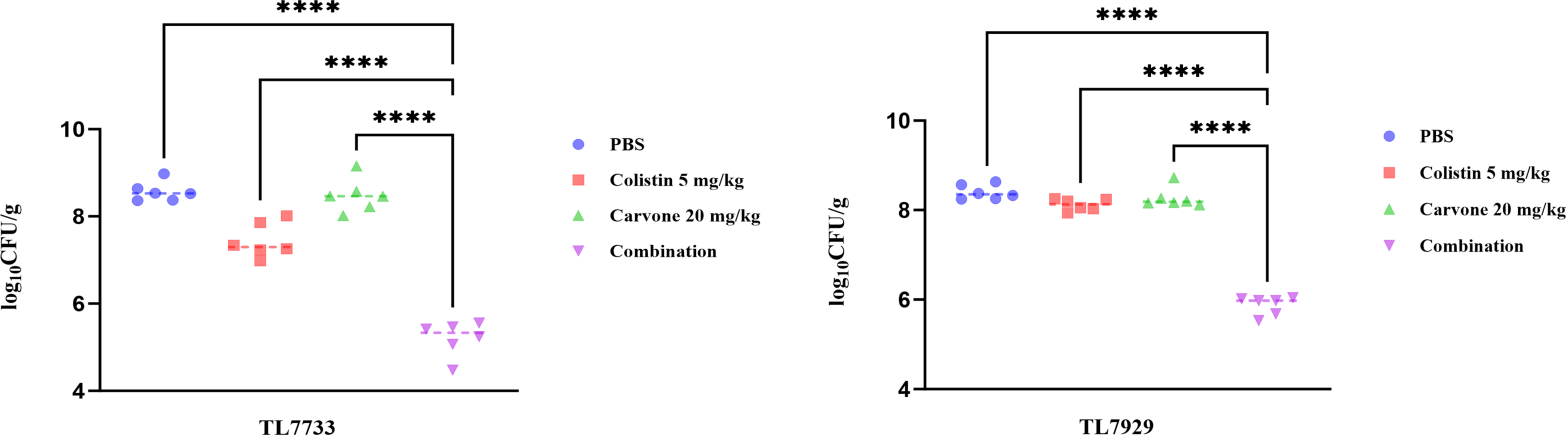
Bacterial colony counts in the neutropenic mouse thigh infection model. Quantitative analysis of the bacterial load in the thigh regions of mice infected with TL7733 and TL7929 after treatment with COL and carvone alone or in combination for 24 h (Δlog10 CFU/g).****P < 0.0001.

We established the neutropenic mouse thigh infection model to evaluate the synergistic effect of *in vivo* antibacterial activity of carvone and COL. Mice were divided into four groups: the phosphate-buffered saline (PBS) group, COL monotherapy group, carvone monotherapy group, and the combination group. The results (Fig. 2) indicated that the bacterial load in thigh tissue was markedly decreased in the combination treatment group versus the PBS group and carvone monotherapy group, with a reduction exceeding 2 log_10_ CFU/mL compared to the single-drug COL group (*P* < 0.05). This indicates that the combined application of carvone and COL exhibits antibacterial effects *in vivo* and possesses significant potential for clinical application.

### Evaluation of biofilm formation inhibition using crystal violet staining assay

**Fig 3:**
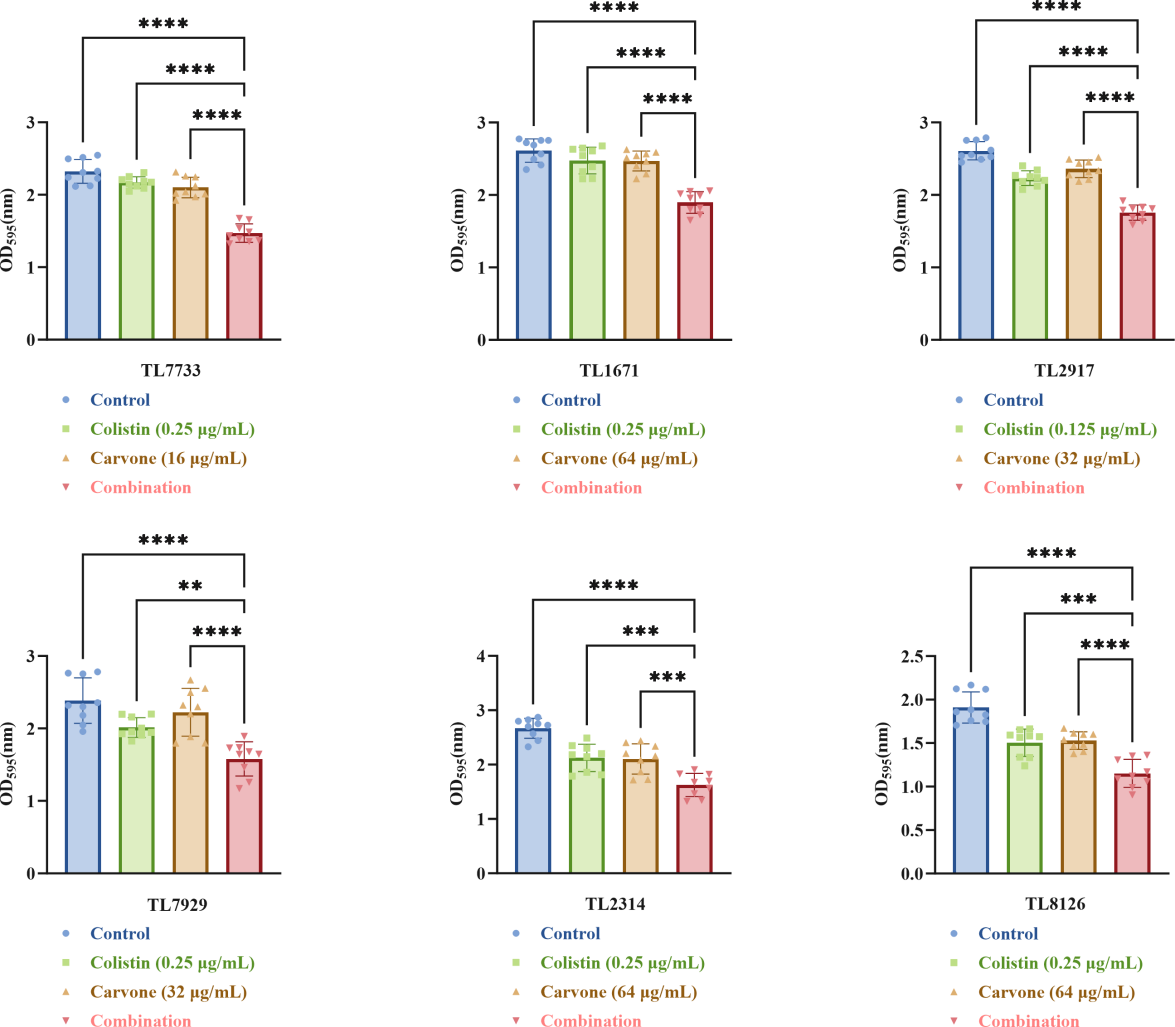
The crystal violet staining assay. The concentrations of both drugs were at sub-inhibitory levels. TL stands for *P. aeruginosa*. **P < 0.01; ***P < 0.001; ****P < 0.0001.

According to the results of the checkerboard assay, the sub-inhibitory concentration of drugs (0.5 × MIC_COL/Carvone in combination_) was selected for the experiment. The results of the growth curve (Fig. S2, the specific experimental procedures are detailed in the Supplementary Information) indicate that, at the sub-inhibitory concentrations, bacterial growth remains largely unaffected, which eliminates the possibility that the inhibition of biofilm formation is due to a direct suppression of bacterial growth. Figure 3 shows that the combined treatment group was more effective in inhibiting biofilm formation in experimental strains compared to single-drug groups (*P* < 0.05).

### Scanning electron microscopy shows inhibition of biofilm formation

**Fig 4:**
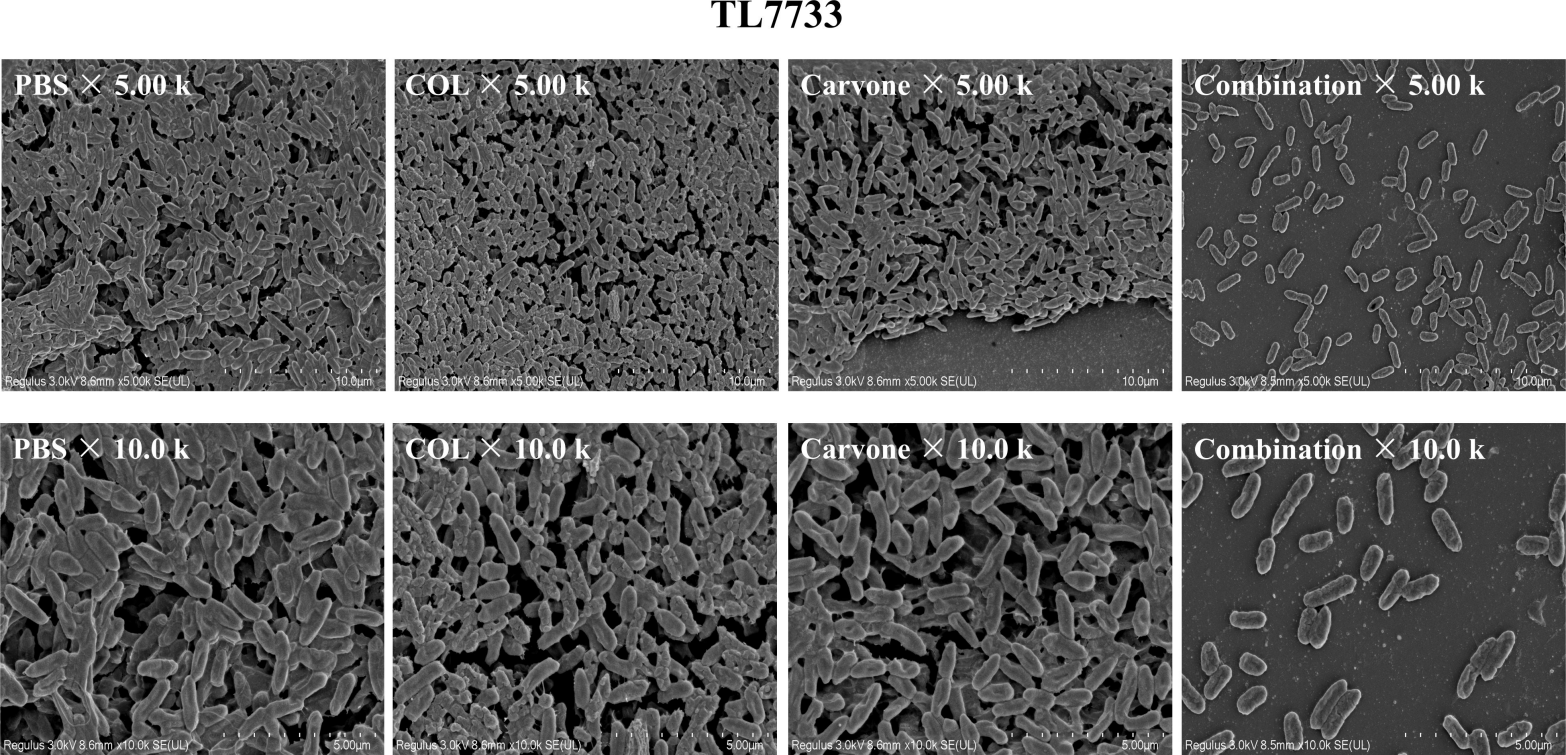
SEM of TL7733 biofilm formation and bacterial morphology. The concentrations of the drugs were all sub-inhibitory, among which the concentration of COL was 0.5 µg/mL, and that of carvone was 16 µg/mL. The magnifications are 5k and 10k, respectively.

The impact of carvone in combination with COL on the biofilm formation of the test strain was further assessed using scanning electron microscopy (SEM). The experimental strain was selected as TL7733, and the drug concentrations were selected at sub-inhibitory levels based on the results obtained from the checkerboard assay. Figure 4 illustrates that in the blank control group, the biofilm structure was compact and spanned the entire visual field. The single-drug groups of COL (0.5 µg/mL) and carvone (16 µg/mL) could form biofilms, and the bacterial quantities were relatively large. However, in the combined treatment group of COL and carvone, the biofilm structure was loose, the bacterial density decreased, and the bacteria were mostly scattered. This finding further confirms that the combination of carvone and COL is highly effective in suppressing biofilm formation.

### Evaluation of biocompatibility

**Fig 5:**
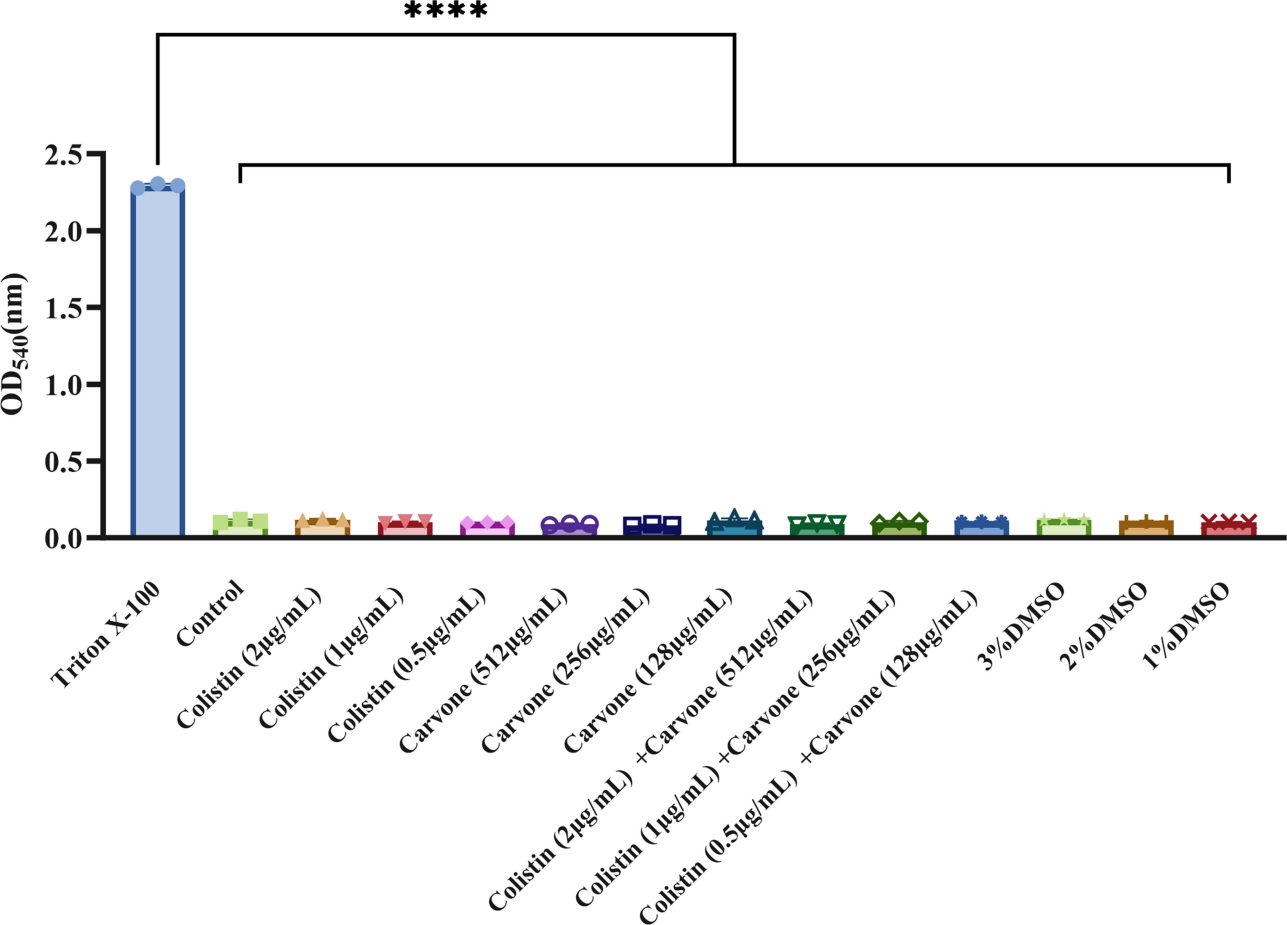
Hemolysis assay. The positive control is 0.1% Triton X-100 solution, and the negative control is the control group treated with PBS. The concentrations of the drugs selected in the figure include all the effective concentrations mentioned in the study. ****P < 0.0001.

We conducted red blood cell hemolysis experiments with healthy male mice red blood cells (RBC) to evaluate the hemolytic activity of carvone, both alone and in combination with COL. The results (Fig. 5) indicate that neither carvone alone (up to 512 µg/mL) nor COL (2 µg/mL) exhibited hemolytic activity. Additionally, the combination of carvone (512 µg/mL) and COL (2 µg/mL) did not show significant hemolytic activity. The concentrations used in the *in vitro* antibacterial study were all lower than this combined concentration, suggesting that the carvone-COL combination is safe for treatment.

**Fig 6:**
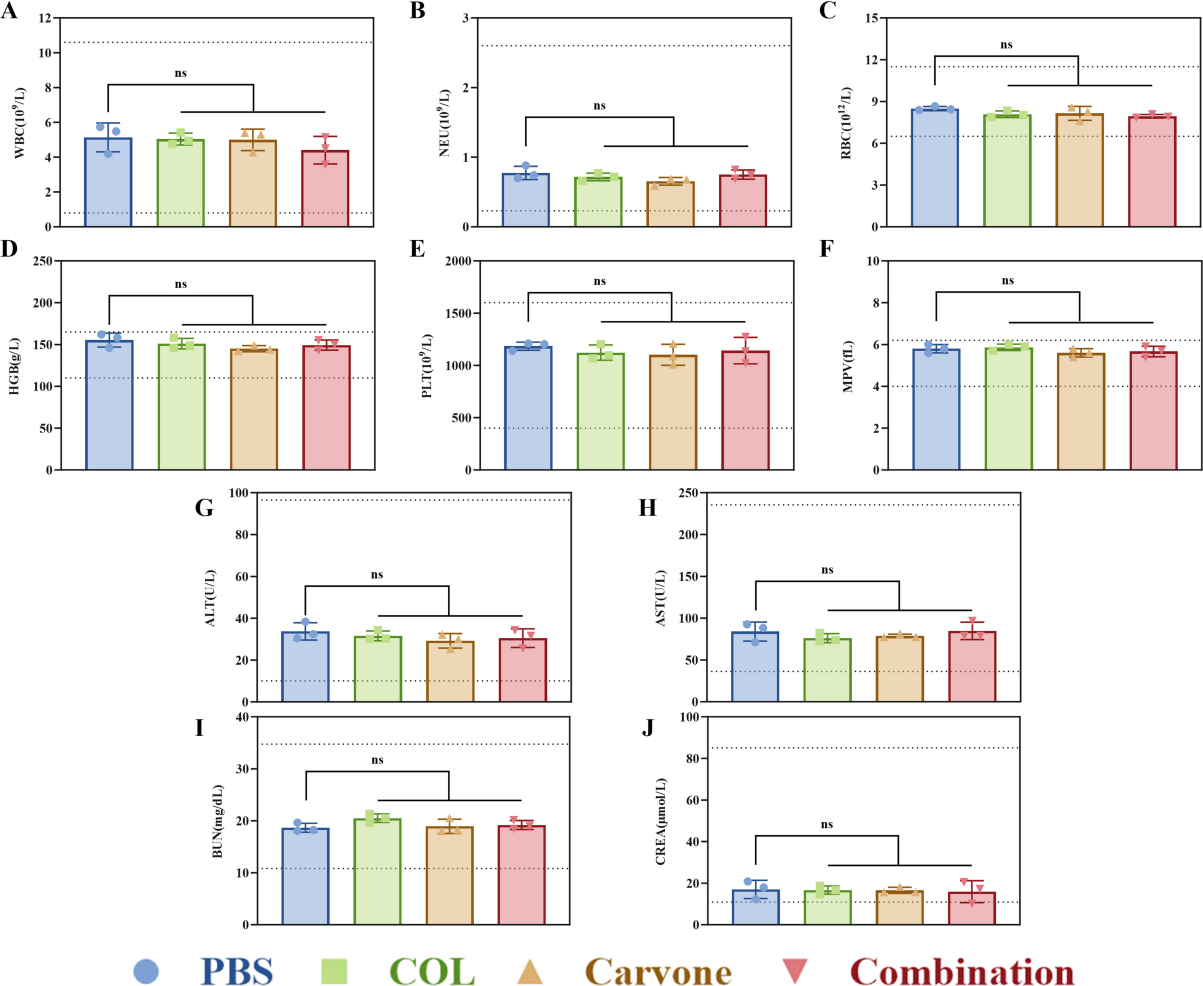
Blood routine and biochemical tests. (**A–F**) Blood routine counts; (**G–J**) biochemical tests; (**G–H**) biochemical indicators for liver function; (**I–J**) biochemical indicators for renal function. The dashed lines in the figure indicate the upper and lower limits of the normal reference range. Drug concentration, COL: 5 mg/kg/24 h, carvone: 20 mg/kg/24 h. ns, *P* > 0.05.

**Fig 7:**
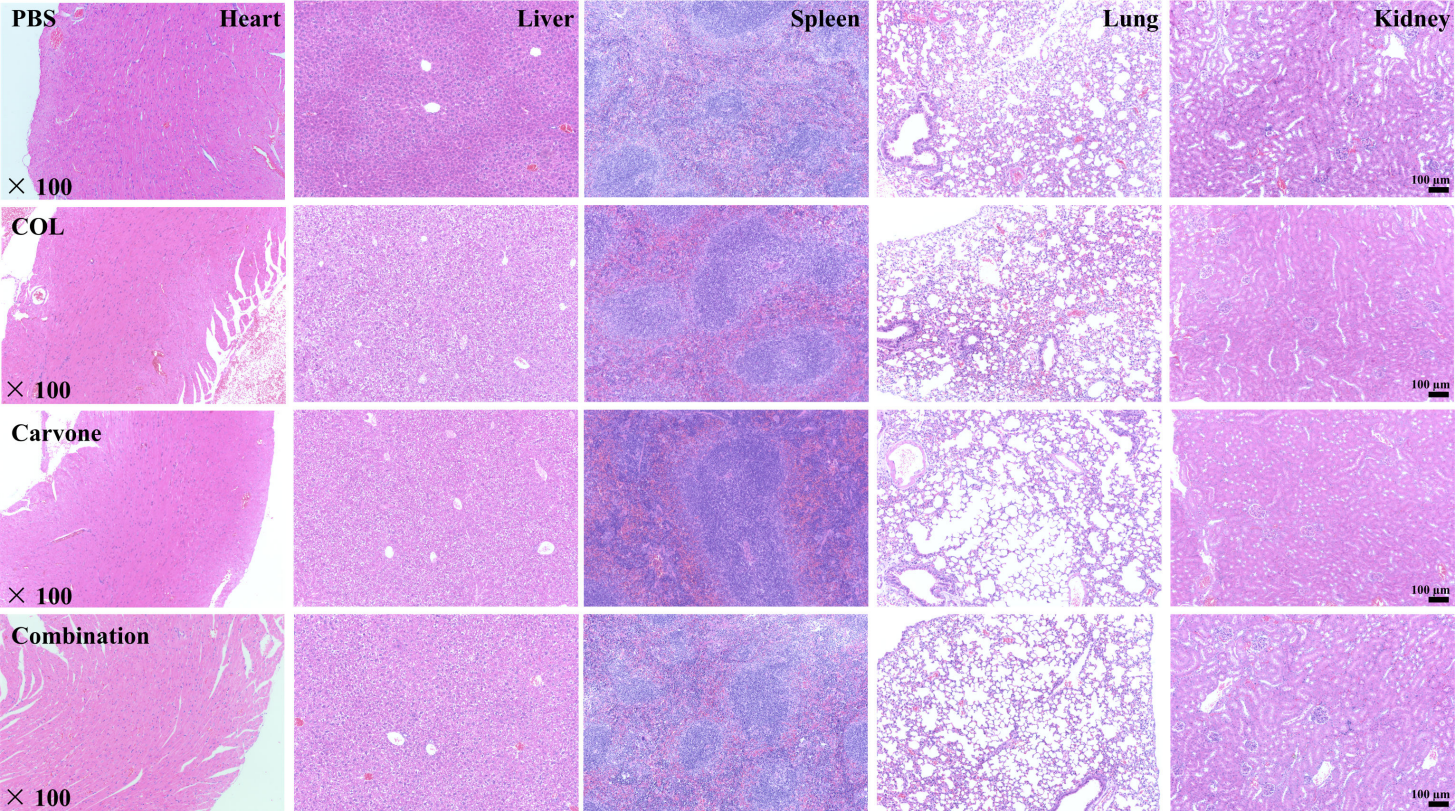
HE staining images of main organs taken from mice. Histological analysis of heart, liver, spleen, lungs, and kidneys in mice. Drug concentration, COL: 5 mg/kg/24 h, carvone: 20 mg/kg/24 h. Scale bar, 100 µm. The magnification is 100 times.

To further evaluate the long-term *in vivo* safety of this treatment combination, we conducted blood routine and biochemical tests on mice after continuous administration for 7 days (carvone at 20 mg/kg/24 h and COL at 5 mg/kg/24 h, consistent with the doses used in the *in vivo* antimicrobial activity studies), as well as histological analyses of the major organs (heart, liver, spleen, lung, and kidney). The results of the blood routine and biochemical indices (focusing on the assessment of liver and renal functions) showed that there were no significant differences in the parameters between the treatment group and the PBS group, and all values were within the normal reference range (Fig. 6) (*P* > 0.05). These indicate that the combined treatment of carvone and COL in mice has a certain level of safety for their hematological system and does not damage their liver and kidney functions. As shown in Fig. 7, no obvious organ damage, abnormalities, or inflammation were found in the hematoxylin and eosin (HE) staining, indicating that carvone combined with COL is harmless to the organ tissues of mice and does not cause excessive inflammatory responses. In conclusion, carvone combined with COL exhibits good biocompatibility *in vivo*, making it a promising candidate for potential *in vivo* applications.

### Study on synergistic antibacterial mechanisms

**Fig 8:**
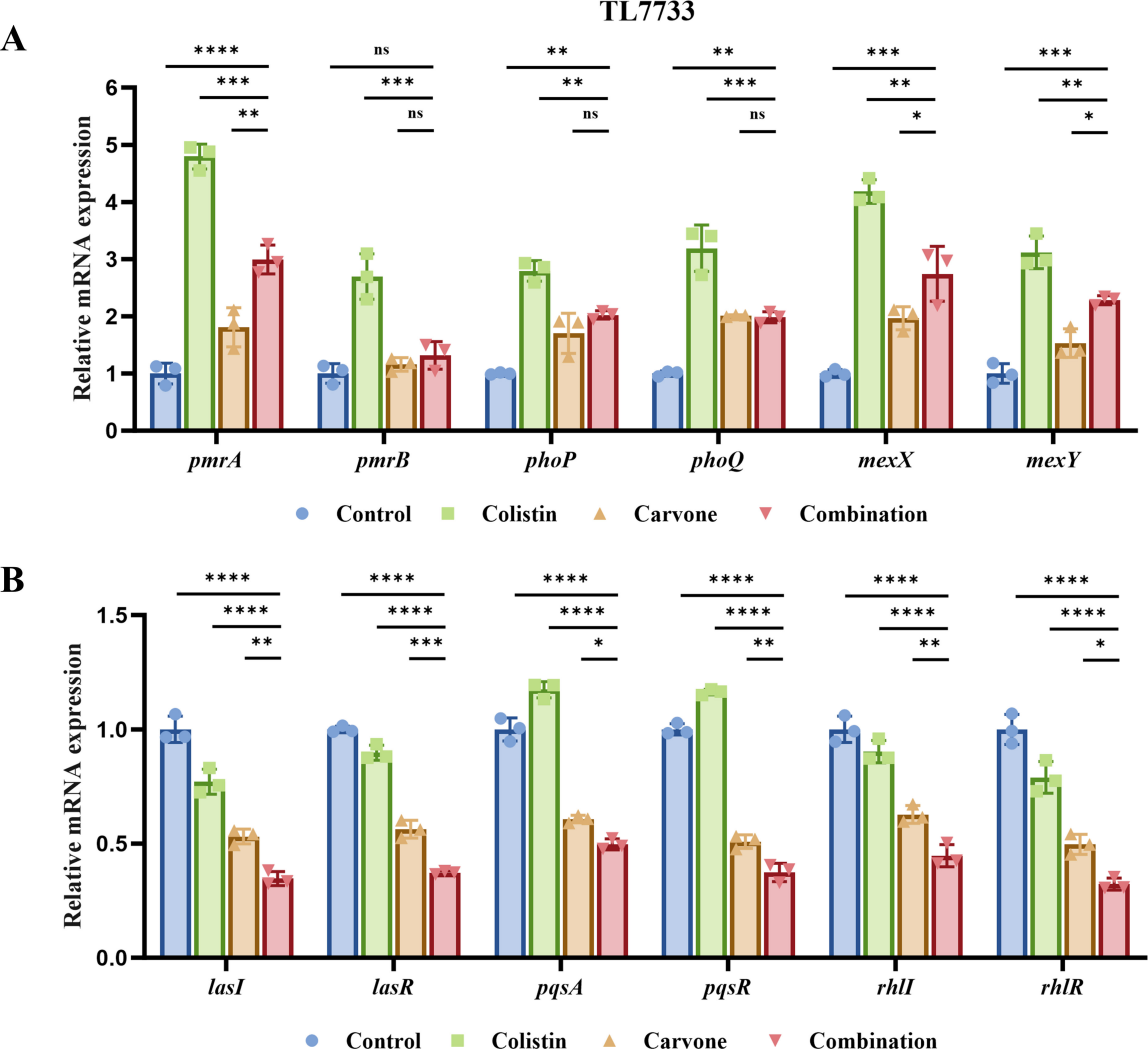
Measurement of mRNA expression level using RT-qPCR. The randomly selected experimental strain TL7733 was treated with different drugs at sub-inhibitory concentrations. This group was divided into four subgroups: PBS, COL (0.25 µg/mL), carvone (32 µg/mL), and combination treatment. (**A**) Expression levels of genes related to LPS modification and efflux pumps. (**B**) Expression levels of quorum-sensing genes. *P < 0.05; **P < 0.01; ***P < 0.001; ****P < 0.0001; ns, *P* > 0.05.

Through crystal violet staining and SEM, we have demonstrated that the combined treatment effectively inhibits *P. aeruginosa* biofilm formation. In addition, the modification of LPS (PmrAB and PhoPQ two-component systems) and the overexpression of efflux pumps (MexXY/OprM) are also important mechanisms of colistin resistance in *P. aeruginosa* (19, 20). Therefore, we randomly selected TL7733 as the experimental strain and treated it with sub-inhibitory concentrations (referencing the checkerboard assay results) of different drugs. Subsequently, reverse transcription-quantitative polymerase chain reaction (RT-qPCR) was used to detect the relative expression levels of genes related to lipopolysaccharide modification, efflux pumps, and quorum sensing. Figure 8A shows that COL monotherapy significantly upregulates the expression of the two-component regulatory genes *pmrAB* and *phoPQ*, as well as the efflux pump genes *mexXY* (*P* < 0.05). Compared with the control group, carvone monotherapy generally results in a modest upregulation of these genes. Existing research indicates that, when faced with external environmental pressures, such as drug exposure, Gram-negative bacteria commonly employ modifications to LPS and the upregulation of efflux mechanisms as a self-protective strategy (21). Accordingly, we hypothesize that carvone, functioning as an environmental stress signal, may also partially trigger the stress response pathways in *P. aeruginosa*, thereby leading to the observed upregulation of the aforementioned genes. Although the combined treatment group also upregulated the expression of these genes, the extent of increase was smaller compared to COL monotherapy (*P* < 0.05). This suggests that these mechanisms are not the primary contributors to the synergistic antibacterial effect. The expression of quorum-sensing genes is closely associated with biofilm formation (22). Figure 8B demonstrates that the combination of carvone and COL significantly reduces the expression of quorum-sensing genes in the experimental strain (*P* < 0.05). This further provides genetic evidence supporting the synergistic antibacterial mechanism through the inhibition of biofilm formation.

**Fig 9:**
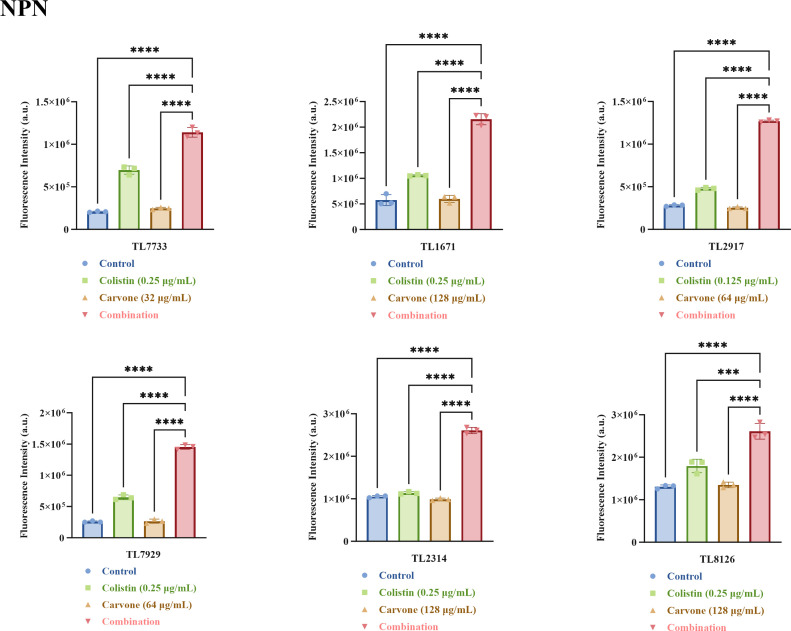
The outer membrane permeability assay. An increase in fluorescence intensity corresponded to a higher degree of membrane permeability. The drug concentrations tested were all sub-inhibitory. NPN stands for NPN dye. TL stands for *P. aeruginosa*. ***P < 0.001; ****P < 0.0001.

**Fig 10:**
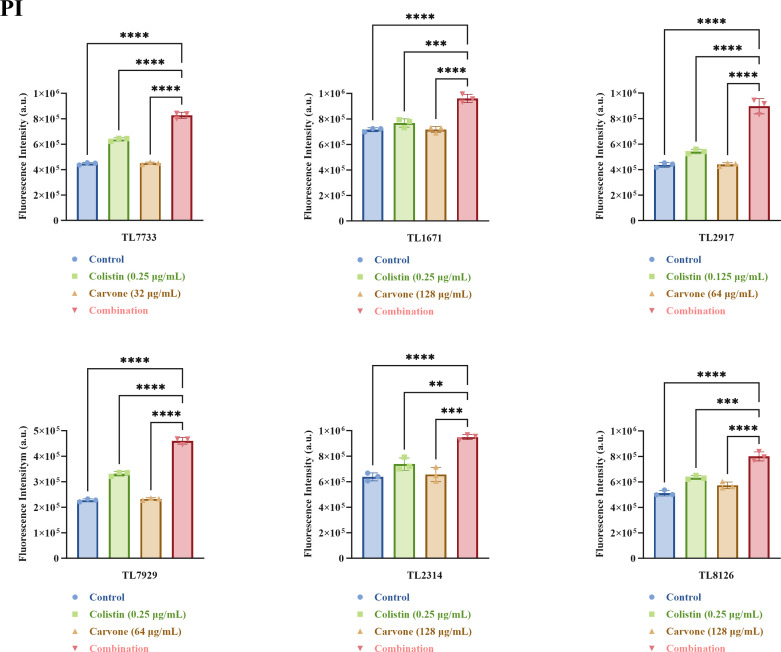
The inner membrane permeability assay. An increase in fluorescence intensity corresponded to a higher degree of membrane permeability. The drug concentrations tested were all sub-inhibitory. PI stands for PI dye. TL stands for *P. aeruginosa*. **P < 0.01; ***P < 0.001; ****P < 0.0001.

Given that the formation of biofilms and low membrane permeability are the main mechanisms for antibiotic resistance of *P. aeruginosa* (22, 23), we also investigated the impact of the combination of carvone and COL on the membrane permeability of *P. aeruginosa*. We further evaluated the synergistic antibacterial mechanism by detecting the permeability of the inner and outer membranes of the experimental strain at sub-inhibitory concentrations (to prevent false positives caused by the direct bacteriostatic or bactericidal effects of excessively high drug concentrations) using propidium iodide (PI) and N-phenyl-1-naphthylamine (NPN) dyes. NPN, a hydrophobic dye, fluoresces upon entering cells with damaged outer membranes (24). As shown in Fig. 9, the combined treatment group exhibited significantly higher fluorescence intensity than the single-drug groups and control (*P* < 0.05), indicating increased outer membrane permeability. PI, a DNA-binding dye, only fluoresces when the inner membrane is compromised, allowing it to enter the cell (25). Figure 10 shows that the combined treatment group had notably higher fluorescence intensity (*P* < 0.05), suggesting enhanced inner membrane permeability.

**Fig 11:**
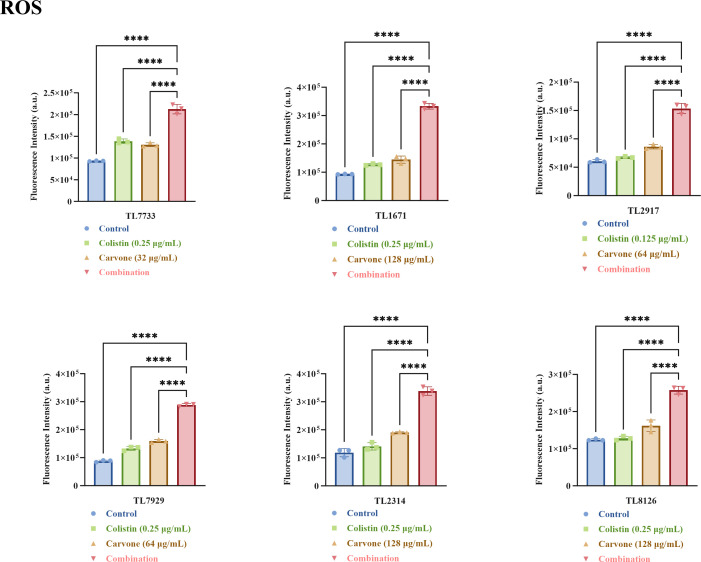
Quantification of ROS. An increase in fluorescence intensity corresponds to an elevation in ROS production. The drug concentrations tested were all sub-inhibitory. ROS stands for reactive oxygen species. TL stands for *P. aeruginosa*. ****P < 0.0001.

Additionally, studies have shown that carvone can induce the generation of reactive oxygen species (ROS) in certain tumor cells, and ROS also play a crucial role in many antibacterial strategies (13, 26). Excessive ROS production can disrupt cellular redox homeostasis and induce oxidative stress, leading to cellular damage (26). Therefore, we further investigated the synergistic antibacterial mechanisms by measuring the production of ROS in bacteria treated with sub-inhibitory concentrations (referencing the checkerboard assay results) of different drugs. The results revealed that the combined treatment group significantly increased the generation of ROS in bacteria (*P* < 0.05) (Fig. 11). It is noteworthy that, compared to the control group, the ROS levels in the carvone monotherapy group also exhibited a general increase, albeit not as pronounced as in the combination therapy group. This suggests that carvone monotherapy may induce oxidative stress in bacteria, an effect that appears to be further amplified when combined with COL.

Collectively, these findings suggest that the synergistic antibacterial action of carvone and COL may be associated with the inhibition of biofilm formation, increased ROS generation, and enhanced membrane permeability. Table S5 indicates that the synergistic effect also occurs in colistin-susceptible *P. aeruginosa*, suggesting that these synergistic mechanisms are not specific to COL-R strains but are probably the general mechanisms of increased uptake.

## DISCUSSION

With the rise of MDR bacteria, COL has been reintroduced for clinical therapy. Nevertheless, an escalating number of COL-R strains, including *P. aeruginosa*, have emerged posing significant clinical challenges (27). Moreover, COL itself possesses nephrotoxicity and neurotoxicity, rendering the notion of increasing the dosage of colistin to combat related resistant strains impracticable (5). Hence, combining COL with other drugs for the treatment of COL-R strains constitutes an economical and effective solution.

*P. aeruginosa* is an opportunistic pathogen responsible for illness and death in cystic fibrosis patients and those with weakened immune systems (28). The WHO has identified *P. aeruginosa* as one of the top three bacteria requiring urgent development of new antibiotics (2). Recent studies have identified several key mechanisms responsible for COL resistance in *P. aeruginosa*. These mechanisms include the reduction of negative charge on the cell membrane surface, alteration of the O-polysaccharide antigen, decreased phospholipid levels, changes in cell membrane permeability, and biofilm formation (10, 23). In simple terms, these mechanisms confer resistance by modifying the cell membrane and forming biofilms to reduce the contact between COL and bacteria. Thus, enhancing membrane permeability and inhibiting biofilm formation are critical for treating COL-R *P. aeruginosa* infections.

Carvone is a monoterpene present in some plant essential oils. Numerous studies have demonstrated that carvone possesses excellent pharmacological properties, including antibacterial and neuroprotective activities (16), and relevant reports have indicated that carvone can be utilized to combat infections caused by *P. aeruginosa* (13). Hence, we endeavored to combine carvone with COL for the treatment of COL-R *P. aeruginosa*.

This study reports the initial synergistic antibacterial activity of carvone and COL against COL-R *P. aeruginosa*. Drug susceptibility testing revealed that 7 of the 12 *P. aeruginosa* strains were multidrug-resistant, and carvone alone showed no significant antimicrobial activity. Checkerboard assays demonstrated that carvone restored the susceptibility of all 12 *P. aeruginosa* strains to COL, reducing the MIC of COL below the susceptibility breakpoint (2 µg/mL) (29). Time-kill assays further confirmed the synergistic effect. Initially, the inhibitory effect of the combined treatment was comparable to COL monotherapy, but after 6 h, COL monotherapy failed to suppress bacterial growth, while the combined treatment continued to inhibit growth, suggesting that carvone extends the bactericidal activity of COL. However, the antibacterial effect of the combined treatment also began to decline by 12 h, with bacterial regrowth observed in some strains (TL7733 and TL2917) as their counts started to increase. This suggests that a second dose could be administered after 12 h for future clinical applications.

Additionally, we established a neutropenic mouse thigh infection model to evaluate the *in vivo* synergistic antibacterial efficacy of carvone and COL. Results demonstrated that the combination significantly reduced bacterial counts in the muscle tissue. Given that biofilm formation is a key mechanism of *P. aeruginosa* antibiotic resistance, we assessed the impact of sub-MIC concentrations of the combined treatment on biofilm inhibition using crystal violet staining and SEM. Crystal violet staining revealed that the combined treatment effectively suppressed biofilm formation, a finding corroborated by SEM analysis. SEM images showed that the biofilm structure in the combined treatment group was loose, with reduced bacterial density, unlike the dense biofilms observed in the single drug and control groups. Bacterial biofilms are often the product of quorum-sensing activation (22). Therefore, we also investigated the changes in the expression levels of quorum-sensing genes in the experimental strains treated with sub-inhibitory concentrations of different drugs using RT-qPCR. The results revealed that carvone alone could reduce the expression of quorum-sensing genes, which is consistent with previous studies (22). Furthermore, the combined treatment of carvone and COL resulted in a more significant decrease in the expression of quorum-sensing genes. This further demonstrates the inhibitory effect of carvone in combination with COL on the biofilm formation of COL-R *P. aeruginosa*. Overall, carvone in combination with COL significantly inhibits biofilm formation by COL-R *P. aeruginosa*.

The primary target of COL is the outer membrane of GNB (30). Modifications of LPS on the bacterial outer membrane and the overexpression of efflux pumps often lead to resistance to COL (19, 20). Therefore, we initially aimed to explore the synergistic antibacterial mechanism by detecting changes in the expression levels of related genes in experimental strains treated with sub-inhibitory concentrations of carvone and COL using RT-qPCR. The results showed that, compared to the COL-alone group, the increase in the expression levels of genes related to LPS modification and efflux pumps was significantly reduced in the carvone and COL combination treatment group. However, compared to the PBS control group, the expression levels of these genes still exhibited an increase. To further investigate the synergistic antibacterial mechanism of carvone and COL, we conducted NPN and PI experiments. The results indicated that the combination therapy significantly enhanced the permeability of both the bacterial inner and outer membranes. The increased membrane permeability could potentially counteract known resistance mechanisms in COL-R *P. aeruginosa*, including LPS modification. This alteration may facilitate improved cellular penetration of COL, which could, in turn, contribute to its enhanced antibacterial efficacy.

Furthermore, we investigated the synergistic antibacterial mechanism by measuring the generation of ROS. The results revealed that the combination of carvone and COL significantly increased ROS production in the experimental strains. The accumulation of ROS can induce oxidative stress in bacteria, causing cellular damage through multiple pathways. This includes attacking the cell membrane, thereby further compromising its integrity (31). Finally, we also discovered that the synergistic antibacterial effect of carvone and COL can also occur in colistin-sensitive *P. aeruginosa*. This suggests that these synergistic mechanisms may be the general mechanisms of increased uptake.

To assess the safety of this combined therapy, we tested its biocompatibility through hemolysis experiments, histopathological analyses, blood routine, and biochemical tests. Results showed that carvone and COL did not induce hemolysis at effective concentrations in combination therapy, nor did they damage tissue or cause inflammatory cell infiltration *in vivo*. This suggests that the combination of carvone and COL significantly reduced the effective dose of COL and avoided the nephrotoxicity associated with high doses of COL. Additionally, carvone has been shown to possess neuroprotective effects (16), potentially alleviating COL’s neurotoxic side effects when combined. Carvone’s anti-cancer and anti-inflammatory properties also indicate significant clinical potential for its combination with COL. This study also found that the combination of carvone and COL exhibits synergistic antibacterial effects against COL-R *Escherichia coli* and *Klebsiella pneumoniae*. However, this synergistic effect is more pronounced against COL-R *P. aeruginosa* (the MIC of COL can be reduced below the clinical breakpoint), which may be related to the significant reduction in the expression of quorum-sensing genes in *P. aeruginosa* by the combination of carvone and COL. We also attempted to combine carvone with antibiotics commonly used clinically against *P. aeruginosa* (ciprofloxacin and tobramycin), but no synergistic effect was observed. This may be because the primary resistance mechanisms of ciprofloxacin and tobramycin (mutations in target enzymes and production of modifying enzymes) cannot be as effectively circumvented by enhancing bacterial membrane permeability through the combination with carvone, unlike the primary resistance mechanism of COL (modification of LPS on the bacterial outer membrane) (19, 32, 33). While our findings are promising, several challenges remain before carvone-COL combination therapy can be widely adopted in clinical settings. Future studies should expand the diversity of experimental strains and animal models (such as chronic infection models) to better mimic the complexity of human infections. Additionally, large-scale clinical trials are needed to confirm the safety and efficacy of this combination. And further experiments are required to obtain pharmacokinetics/pharmacodynamics or resistance-evolution data.

### Conclusion

This research discovered that carvone can restore the COL susceptibility of COL-R *P. aeruginosa*, lower the dose of COL, and thereby decrease the clinical side effects related to COL. Furthermore, the combination of carvone and COL effectively suppressed the formation of *P. aeruginosa* biofilms. This study might offer a novel clinical therapeutic approach for COL-R *P. aeruginosa* infections and merits further research.

## MATERIALS AND METHODS

### Processes common to all experiments

All experiments in this study were performed with at least three technical and biological replicates. All experimental strains involved in the study were primarily selected based on a randomization principle, with additional consideration given to the diversity of strain backgrounds to ensure a representative selection. The selection of drug concentrations for each experiment was rigorously determined based on the results of the checkerboard assay.

### Strain sources and preservation

Every clinical strain utilized in this investigation came from patients at the First Affiliated Hospital of Wenzhou Medical University. The selected strains are COL-R *P. aeruginosa* isolated between 2015 and 2018. The clinical information of the strains can be found in Table S1 in the Supplementary Information. The primary inclusion criterion was COL-R *P. aeruginosa* (defined as MIC ≥ 4 µg/mL). Strains with unstable MIC values, intermediate susceptibility (2 µg/mL ≤ MIC < 4 µg/mL), or sensitivity (MIC < 2 µg/mL), as well as those exhibiting heteroresistance, were excluded. For comparison, *P. aeruginosa* ATCC27853, which was supplied by the National Center for Clinical Laboratories, was used as a quality control strain. The strains were kept in Luria-Bertani (LB) broth with 30% glycerol at −80°C for upcoming studies after being described using matrix-assisted laser desorption/ionization time-of-flight mass spectrometry (MALDI-TOF MS; bioMérieux, Lyon, France).

### Chemical reagents and antibiotics

(S)-(+)-Carvone was procured from MedChemExpress LLC, NJ, USA, and dissolved in dimethyl sulfoxide (DMSO) (Sigma-Aldrich, Saint Louis, MO, USA). At the effective antibacterial concentrations used in subsequent experiments, the DMSO content was maintained below 1% (DMSO at this content has no antibacterial activity) (34, 35). All the antibiotics involved in this study were acquired from Kangtai Biotech Co., Ltd. in Wenzhou, Zhejiang, China.

### Determination of drug sensitivity

The microbroth dilution technique was employed to determine the MICs of each drug against the experimental strain, following a method consistent with previous studies (36, 37). Cation-adjusted Mueller-Hinton broth (CAMHB) was added to 96-well plates, followed by sequential drug dilution to achieve 100 µL of drug-containing broth per well. Subsequently, 100 µL of the test strain (1.5 × 10^6^ CFU/mL) was added and incubated at 37°C for 16 to 18 h. Visual inspection identified the lowest drug concentration inhibiting bacterial growth as the MIC. Drug susceptibility was evaluated using 2024 CLSI guideline breakpoints, with strains resistant to three or more antimicrobial categories designated as MDR strains. Detailed procedures can be found in the Supplementary Information. The MICs of carvone against the experimental strains were compared in both CAMHB and LB broth, revealing no major differences (Table S6). This finding provides a foundational basis for subsequent experiments involving the use of LB broth.

### Checkerboard assay

The synergistic effect between carvone and COL was assessed using a checkerboard assay, following the methodology outlined in prior studies (38). Briefly, the two compounds were serially diluted and combined in a 96-well plate to create various concentration gradients. Bacterial inoculation, incubation, and MIC result interpretation followed the same procedures as previously described for drug sensitivity testing.

The FICI was calculated to assess the synergistic interaction between the two drugs in combination (39). The relevant calculation formula is presented as follows: FICI = FIC_Carvone_ + FIC_COL_ = (MIC_Carvone in combination_/MIC_Carvone alone_) + (MIC_COL in combination_/MIC_COL alone_). FICI ≤ 0.5 suggests a synergistic effect, 0.5 ˂ FICI ≤ 4 suggests no interaction, and FICI > 4 suggests an antagonistic effect.

### Time-kill assay

Six strains were selected as representative experimental strains based on a randomization principle. The specific methodology is comparable to that reported in the previous study (40). The tested strains were inoculated onto Columbia blood agar plates and incubated at 37°C for 16–18 h. First, the tested strains on the blood agar plates were adjusted to a turbidity equivalent to 0.5 McFarland standard (1.5 × 10⁸ CFU/mL) using 0.9% sodium chloride. Then, 200 μL of the bacterial suspension was taken and added to 20 mL of LB broth (diluted 100-fold, with an initial concentration of approximately 10⁶ CFU/mL). COL and carvone were individually or jointly added to the LB broth containing bacteria as previously described, and the groups were divided into the COL monotherapy group, the carvone monotherapy group, and the combination therapy group. In the control group, an equivalent volume of LB broth was added to the bacterial suspension in LB broth. The final concentrations of the drugs were determined according to the results of the checkerboard assay (the concentrations of COL and carvone were 3 × MIC_COL in combination_ and 3 × MIC_Carvone in combination_, respectively). Subsequently, the resulting mixture was incubated with shaking at 37°C and 200 rpm. Equal volumes of the culture were sampled at six time points: 0, 2, 4, 6, 12, and 24 h. Following serial dilution, 100 µL of the bacterial suspension was spread onto LB agar plates and cultivated at 37°C for 16 to 18 h to allow for colony counting.

### Neutropenic mouse thigh infection model

A neutropenic mouse thigh infection model was constructed to explore the *in vivo* combined bactericidal effect of carvone and COL. The construction of this model was similar to the previous study (41). Neutropenia was induced in mice by intraperitoneal injection of cyclophosphamide at doses of 150 mg/kg 4 days prior to the experiment and 100 mg/kg 1 day before the experiment. TL7733 and TL7929 were randomly selected from the six strains used in the time-kill assay to serve as the representative strains for this experiment. The experimental strains, at a concentration of 1.5 × 10⁵ CFU/mL, were administered at a dose of 100 µL per hind leg, injected directly into the thigh muscle of each mouse. The mice were allocated into four groups: the PBS group, COL monotherapy group, carvone monotherapy group, and the combination group. Subsequently, 200 µL of the drugs was intraperitoneally injected 2 h after infection. Drugs were all administered in a single dose. The dosage of COL was 5 mg/kg, and that of carvone was 20 mg/kg (this dose was selected with reference to previous relevant studies, and carvone at this dose has certain *in vivo* safety) (13, 42). Twenty-four hours post-treatment, the mice were euthanized, and their thigh muscle tissues were dissected. The tissues were then homogenized, and the resulting homogenates were spread onto LB agar plates for bacterial colony counting. In this experiment, male CD-1 (ICR) mice aged 28–34 days were purchased from Vital River in Zhejiang, China. The mice weighed 24–26 g. There were 3 mice in each group, with a total of 12 mice (each group consists of six data points, with each data point representing the bacterial load count in the thigh muscle tissue sample of each mouse’s hind leg). Since each mouse has two hind legs, a single mouse can contribute two data points. Each group includes three mice, resulting in a total of six data points per group. Moreover, each data point is the mean value of three technical replicates. The National Standard for Laboratory Animals of China (GB 14925-2010) was followed for housing the mice. Every experimental method was carried out in accordance with the Wenzhou Guidelines for Laboratory Animal Welfare and Ethics and approved by the Ethics Committee of Wenzhou Medical University’s First Affiliated Hospital (Approval No. SYXK 2021-0017).

### Crystal violet staining assay

To ensure the relevance of the results, the selected experimental strains were consistent with those used in the time-kill assay. The specific research methodology was formulated in accordance with existing literature (43). A suspension of the test strain (1.5 × 10^6^ CFU/mL) was added to the wells of a 96-well plate and co-cultured with COL, carvone, or both at sub-inhibitory concentrations (a measure that avoids the risk of false-positive results due to excessively strong bacteriostatic or bactericidal activity). Each well contained 100 µL of the strain suspension and 100 µL of drug-containing LB broth, with a control group of 100 µL strain suspension and 100 µL pure LB broth. Each group was tested using three replicate wells and was repeated three times biologically. Following a 24-h incubation period at 37°C, the wells were air-dried and twice rinsed with 200 µL of 1× PBS to get rid of any remaining planktonic cells. Following 1% crystal violet staining, 95% ethanol was used to destain the wells. A microplate reader was used to detect absorbance at 595 nm.

### Scanning electron microscopy

To correlate *in vivo* and *in vitro* experiments, TL7733 was randomly selected from the representative strains of the neutropenic mouse thigh infection model. The methodology was primarily referenced from previous literature (44). Sterile silicon chips were placed in a 24-well plate with the polished side facing up. Each well received 500 µL of drug-containing LB broth and 500 µL of bacterial suspension, forming the experimental group. This group was divided into three subgroups: COL (0.5 µg/mL), carvone (16 µg/mL), and combination treatment. The control group received only LB broth without any drugs. After incubation at 37°C for 24 h, the samples were washed twice with sterile PBS and fixed with 2.5% glutaraldehyde (Merck) for 4 h at 4°C. Gradual dehydration was performed using ethanol solutions (30%, 50%, 70%, 90%, and 100%) for 15 min each. The samples were then air-dried in a vacuum desiccator at room temperature and coated with a 5 nm gold layer using an ion sputtering device. SEM analysis was conducted using a Hitachi S3000N (Tokyo, Japan) at 10 kV according to the manufacturer’s instructions.

### Hemolytic assay

In brief, fresh blood from healthy male mice was prepared as a 5% RBC suspension. Equal volumes of this RBC suspension were mixed with varying concentrations of the test drug to achieve a final volume of 1 mL. The negative control consisted of PBS, while the positive control was 0.1% Triton X-100 solution. Following a 2 h incubation at 37°C, the supernatant was separated via centrifugation, and the absorbance was subsequently quantified at a wavelength of 540 nm (45).

### Evaluation of *in vivo* safety

To ensure the *in vivo* safety of the combined administration of carvone and COL, we referred to the literature and collected blood from the orbits of mice after seven consecutive days of administration for blood routine counts and biochemical tests. Then, the mice were sacrificed by cervical dislocation. The heart, liver, spleen, lungs, and kidneys were dissected and removed from them. These organs were made into tissue sections and stained with HE for analysis to evaluate the inflammatory infiltration of various organ tissues (46, 47). Drug concentrations (carvone 20 mg/kg/24 h, COL 5 mg/kg/24 h) and injection method (intraperitoneal injection) were determined according to the protocol established for the neutropenic mouse thigh infection model in this study. The control group received PBS. The detailed procedure can be found in the Supplementary Information.

### Reverse transcription-quantitative polymerase chain reaction

The selection of experimental strains was consistent with the SEM. The methodology was primarily referenced from previous literature (22, 48). In brief, the experimental strain was cultured in LB broth until the optical density at 600 nm reached 0.6–0.8. Following treatment with sub-inhibitory concentrations of various drugs for 4 h, total mRNA was extracted. Reverse transcription was performed using a reverse transcription kit (Applied Biosystems, USA) according to the manufacturer’s instructions to obtain cDNA. RT-qPCR was conducted using SYBR Green (SYBR Green kit, Applied Biosystems, USA) and qPCR primers (Table S7). The experiment was independently repeated three times. *16S rRNA* was used as the reference housekeeping gene, and the expression levels of the target genes were calculated using the comparative threshold cycle (ΔΔCt) method.

### Permeability of the inner and outer membranes of bacteria

The permeability of the bacterial inner and outer membranes was assessed using PI and NPN fluorescent probes, respectively, with the methodology primarily adapted from relevant literature (34). The selected experimental strains were consistent with those used in the time-kill assay. Bacteria were cultured overnight, washed with PBS, and adjusted to an OD_600_ of 0.3–0.4. The bacterial suspensions were exposed to various sub-inhibitory drug concentrations (a measure that avoids the risk of false-positive results due to excessively strong bacteriostatic or bactericidal activity) at 37°C for 2 h, then rinsed twice with PBS. Bacteria were stained with PI (50 µg/mL) and NPN (30 µg/mL) for 30 min to evaluate inner and outer membrane permeability, respectively. Fluorescence intensity was measured using a microplate reader, with PI set at excitation/emission wavelengths of 535 nm/615 nm and NPN at 350 nm/420 nm.

### ROS detection

Based on a previously established method, the production of ROS in bacteria was quantified using a commercial assay kit (34). The selected experimental strains were consistent with those used in the time-kill assay. Briefly, overnight LB bacterial cultures were washed three times with PBS and subsequently diluted with PBS until the OD600 reached a range of 0.3 to 0.4. The fluorescent probe 2′,7′-dichlorodihydrofluorescein diacetate (DCFH-DA) was incubated with the bacteria at a 1:1,000 ratio at 37°C in the dark for 30 min. After successful loading of the probe, the bacteria were washed three times with PBS to remove any unbound probe. Following treatment with subinhibitory concentrations of the drug for 2 h, fluorescence intensity was assessed at an excitation wavelength of 488 nm and an emission wavelength of 525 nm.

### Statistical analysis

The data are shown as the average ± standard deviation (SD) based on a minimum of three separate experiments. Significance was determined using one-way analysis of variance, followed by Tukey’s post hoc test. For all analyses, *P < 0.05; **P < 0.01; ***P < 0.001; ****P < 0.0001; ns, *P* > 0.05. Statistical analyses were performed using GraphPad Prism 10.0 (GraphPad Software, LLC; San Diego, California, USA).

## Data Availability

All original data generated during the study are included in the article as figures and tables. For further inquiries, please contact the corresponding author.

## SUPPLEMENTAL MATERIAL

Supplemental material spectrum.02307-25-s0001.docx Tables S1 to S7; Fig. S1 and S2.

ASM does not own the copyrights to Supplemental Material that may be linked to, or accessed through, an article. The authors have granted ASM a non-exclusive, world-wide license to publish the Supplemental Material files. Please contact the corresponding author directly for reuse.
