# Supplementary material for: Evaluation of the combined efficacy of carvone and colistin against colistin-resistant Pseudomonas aeruginosa: in vitro and in vivo studies
Source: Microbiol Spectr. 2026 May 14;14(6):e02307-25. doi: 10.1128/spectrum.02307-25 (PMC13228088; doi:10.1128/spectrum.02307-25)
Supplement: Supplemental material — Tables S1 to S7; Fig. S1 and S2. [file spectrum.02307-25-s0001.docx]

**Supporting Information for**

**Evaluation of the combined efficacy of carvone and colistin against colistin-resistant *Pseudomonas aeruginosa*: *in vitro* and *in vivo* studies**

Zeyong Zhong^a,b^, Zhexiao Ma^a^, Yichi Zhang^b^, Yanchun Gong^b^, Yunying Ge^b^,

Yuhan Yang^a^, Tieli Zhou^b,*^and Jianming Cao^a,b,*^

^a^School of Laboratory Medicine and Life Science, Wenzhou Medical University, Wenzhou, China

^b^Department of Clinical Laboratory, The First Affiliated Hospital of Wenzhou Medical University; Key Laboratory of Clinical Laboratory Diagnosis and Translational Research of Zhejiang Province

**^*^Corresponding authors**

**Jianming Cao** (Corresponding author). Address: School of Laboratory Medicine and Life Science, Wenzhou Medical University, Wenzhou, China; Tel: +86-0577-8806-9595; Fax: +86-0577-8806-9595; E-mail: [wzcjming@163.com](mailto:wzcjming@163.com).

**Tieli Zhou** (Co corresponding author). Address: Department of Clinical Laboratory, The First Affiliated Hospital of Wenzhou Medical University, Wenzhou, 325000, China; Tel: +86-0577-8668-9885; Fax: +86-0577-8668-9885; E-mail: [wyztli@163.com](mailto:wyztli@163.com).

**Supplementary Information includes:**

- Table S1-7,Figure S1-2 and the detailed procedure of determination of drug sensitivity, the growth curve and evaluation of *in vivo* safety.

**Table S1** The MICs of commonly used clinical antibiotics and Carvone against COL-R *P. aeruginosa*

| **Strains^a^** | **Sequence type** | **Specimen type** | **Antibiotics^b^** | | | | | | | | **Carvone** |
| --- | --- | --- | --- | --- | --- | --- | --- | --- | --- | --- | --- |
|  |  |  | **ATM** | **CAZ** | **FEP** | **IPM** | **CIP** | **LVX** | **TOB** | **COL** |  |
|  |  |  | **Breakpoints (S-R)^c^ MIC (mg/L)** | | | | | | | |  |
|  |  |  | **8-32** | **8-32** | **8-32** | **2-8** | **0.5-2** | **1-4** | **1-4** | **2-4** |  |
| **TL7733** | 1058 | Urine | **32** | 16 | **64** | 2 | 1 | 2 | ≤1 | **8** | >512 |
| TL1671 | 1020 | Exudate | 8 | 4 | 8 | 2 | 0.25 | 1 | 1 | **32** | >512 |
| **TL2917** | 471 | Sputum | **32** | 16 | 16 | **16** | 0.25 | 2 | **8** | **8** | >512 |
| **TL7929** | 244 | Pus | 4 | 2 | **256** | 4 | **8** | ≤0.25 | 1 | **32** | >512 |
| TL2314 | 508 | Sputum | 16 | **32** | 16 | 4 | 0.5 | 2 | 2 | **8** | >512 |
| **TL8126** | 244 | BALF**^d^** | 4 | 16 | **256** | **16** | 0.5 | 0.5 | ≤1 | **32** | >512 |
| **TL7333** | 1971 | Urine | 16 | 4 | 4 | 2 | **2** | **8** | **16** | **16** | >512 |
| TL7508 | 3875 | Blood | 4 | 2 | ≤1 | ≤1 | **4** | 2 | ≤1 | **8** | >512 |
| TL7505 | 1400 | Sputum | 4 | 2 | ≤1 | 2 | ≤0.25 | ≤0.25 | ≤1 | **8** | >512 |
| TL7548 | 357 | Exudate | 4 | 4 | 2 | 2 | 0.5 | 0.5 | ≤1 | **16** | >512 |
| **TL7440** | 508 | Sputum | 16 | **64** | **64** | **8** | **4** | **4** | 0.5 | **16** | >512 |
| **TL8269** | 168 | Sputum | **32** | 8 | 8 | **16** | **4** | **4** | ≤1 | **8** | >512 |

^a^strains in boldface are multidrug-resistant (MDR) strains.

**^b^**ATM, aztreonam; CAZ, ceftazidime; FEP, cefepime; IPM, imipenem; CIP, ciprofloxacin; LVX, levofloxacin; TOB, tobramycin; COL, colistin.

**^c^**S-R represents the susceptible (S) breakpoint to resistant (R) breakpoint, according to CLSI supplement M100 (34th edition) and EUCAST.

**^d^**BALF, bronchoalveolar lavage fluid.

**Table S2** Mutational analysis of the PmrAB, PhoPQ regulatory pathways in COL-R *P. aeruginosa*

| Strains^a^ | Sequence type | PmrB (477 aa)^b^ | PhoQ (448 aa) | MIC(μg/mL)^c^ |
| --- | --- | --- | --- | --- |
| TL7733 | 1058 | Y345H | V260G | 8 |
| TL1671 | 1020 | V15I, P216S |  | 32 |
| TL2917 | 471 | G179D | V260G | 8 |
| TL7929 | 244 |  | V260G | 32 |
| TL2314 | 508 |  | V260G | 8 |
| TL8126 | 244 |  | V260G | 32 |
| TL7333 | 1971 |  | V260G | 16 |
| TL7508 | 3875 |  | V260G | 8 |
| TL7505 | 1400 |  | V260G | 8 |
| TL7548 | 357 |  | V260G, V329E | 16 |
| TL7440 | 508 |  | V260G | 16 |
| TL8269 | 168 |  | V260G | 8 |

^a^For colistin-resistant isolates, the sequences were compared with the colistin-susceptible *Pseudomonas aeruginosa* strain PAO1.

^b^aa, amino acids.

^c^The minimum inhibitory concentration of colistin to strains. The drug breakpoint is 2-4 μg/mL, according to CLSI supplement M100 (34th edition) and EUCAST.

**Table S3** FICI values for colistin/Carvone combinations against colistin-resistant *Escherichia coli* and *Klebsiella pneumoniae*

| **Strains** | **Monotherapy MIC (μg/mL)** | | **Combination MIC (μg/mL)** | | **FICI** | **Interpretation** |
| --- | --- | --- | --- | --- | --- | --- |
|  | **Carvone** | **COL** | **Carvone** | **COL** |  |  |
| *E. coli* |  |  |  |  |  |  |
| DC19144 | >512 | 8 | 64 | 2 | <0.375 | Synergistic |
| DC90 | >512 | 8 | 32 | 2 | <0.313 | Synergistic |
| DC3737 | >512 | 8 | 64 | 2 | <0.375 | Synergistic |
| *K. pneumoniae* |  |  |  |  |  |  |
| FK12716 | >512 | 32 | 128 | 4 | <0.375 | Synergistic |
| FK6696 | >512 | 64 | 128 | 8 | <0.375 | Synergistic |
| FK12771 | >512 | 32 | 64 | 8 | <0.375 | Synergistic |

The fractional inhibitory concentration index (FICI) was computed to assess the synergistic effect of the combination of two drugs.

FICI = FIC_Carvone_+ FIC_COL_ = (MIC_Carvone in combination_ / MIC_Carvone alone_) + (MIC_COL in combination_ / MIC_COL alone_).

FICI ≤ 0.5 indicates a synergistic effect, 0.5 ˂ FICI ≤ 4 indicates no interaction, and FICI > 4 indicates an antagonistic effect.

**Table S4** Checkerboard assay results of carvone combined with ciprofloxacin or tobramycin against relevant resistant *P. aeruginosa*

| **Strains** | **Monotherapy MIC (μg/mL)** | | **Combination MIC (μg/mL)^d^** | | **FICI^c^** | **Interpretation** |
| --- | --- | --- | --- | --- | --- | --- |
|  | **Carvone** | **CIP^a^** | **Carvone** | **CIP^b^** |  |  |
| TL7929 | >512 | 8 | 16 | 4 | 0.5000< FICI <0.531 | No interaction |
| TL7333 | >512 | 2 | 8 | 1 | 0.5000< FICI <0.516 | No interaction |
| TL7508 | >512 | 4 | 8 | 2 | 0.5000< FICI <0.516 | No interaction |
| TL7440 | >512 | 4 | 8 | 2 | 0.5000< FICI <0.516 | No interaction |
| TL8269 | >512 | 4 | 16 | 2 | 0.5000< FICI <0.531 | No interaction |
|  | **Carvone** | **TOB** | **Carvone** | **TOB** |  |  |
| TL2917 | >512 | 8 | 8 | 4 | 0.5000< FICI <0.516 | No interaction |
| TL7333 | >512 | 16 | 8 | 8 | 0.5000< FICI <0.516 | No interaction |

^a^CIP, ciprofloxacin. ^b^TOB, tobramycin.

^c^The fractional inhibitory concentration index (FICI) was computed to assess the synergistic effect of the combination of two drugs(A and B). FICI = FIC_A_+ FIC_B_ = (MIC_A in combination_ / MIC_A alone_) + (MIC_B in combination_ / MIC_B alone_). FICI ≤ 0.5 indicates a synergistic effect, 0.5 ˂ FICI ≤ 4 indicates no interaction, and FICI > 4 indicates an antagonistic effect.

^d^The principle for selecting combination well for calculation is that the well must appear clear to the naked eye, indicating an absence of significant bacterial growth. Additionally, the concentrations of the two drugs in this well should be the lowest possible among all clear wells. This approach ensures that FICI is minimized, providing a more reliable assessment of potential synergistic interactions between the two drugs. The principle of selection can be understood more intuitively with reference to Figure S1.


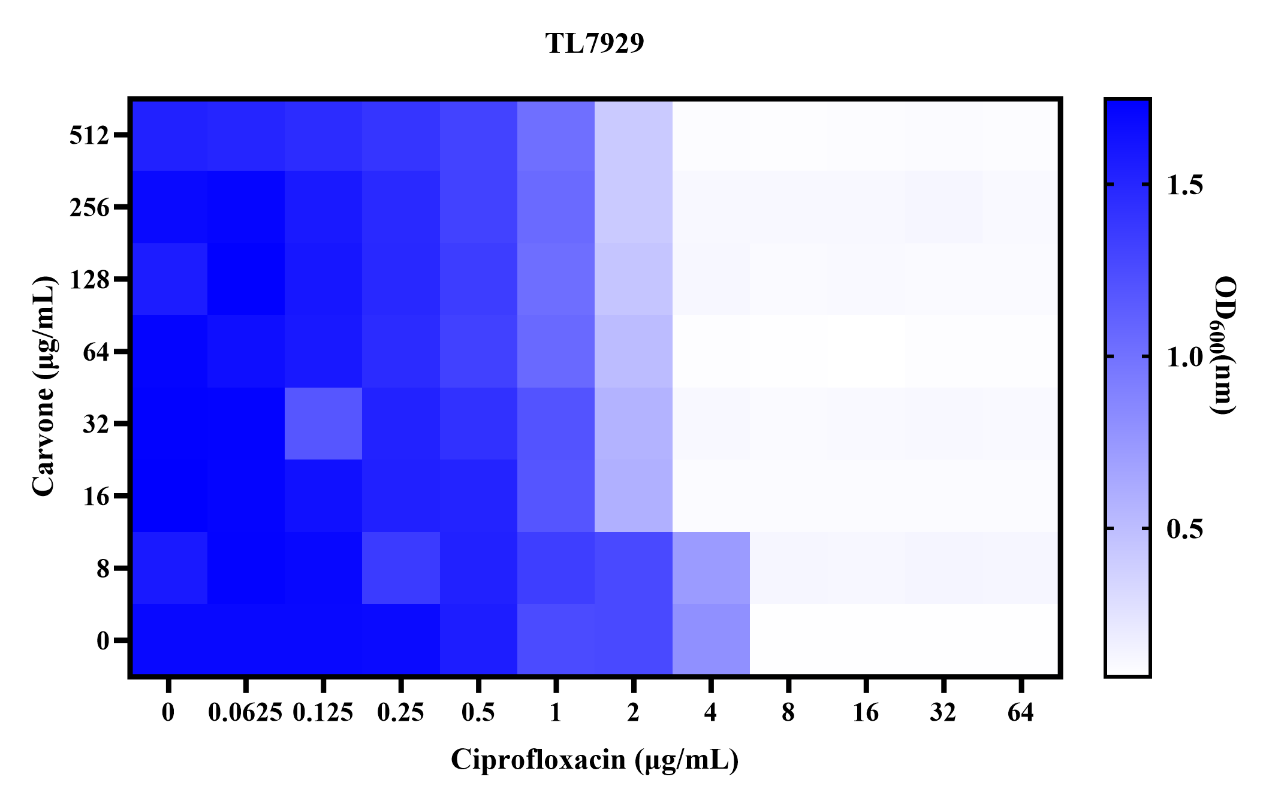


**Figure S1** Checkerboard assay results of carvone combined with ciprofloxacin against *P. aeruginosa* TL7929. The well circled in red on the figure is selected for the calculation of the FICI value. At this point, the FICI value is minimized, making it the most suitable metric for determining whether the two drugs exhibit a synergistic effect.

**Table S5** FICI values for colistin/Carvone combinations against colistin-susceptible *P. aeruginosa*

| **Strains** | **Monotherapy MIC (μg/mL)** | | **Combination MIC (μg/mL)** | | **FICI** | **Interpretation** |
| --- | --- | --- | --- | --- | --- | --- |
|  | **Carvone** | **COL** | **Carvone** | **COL** |  |  |
| TL3567 | >512 | 0.25 | 16 | 0.0625 | <0.281 | Synergistic |
| TL3579 | >512 | 0.5 | 16 | 0.125 | <0.281 | Synergistic |
| TL3670 | >512 | 0.125 | 32 | 0.03125 | <0.313 | Synergistic |

The fractional inhibitory concentration index (FICI) was computed to assess the synergistic effect of the combination of two drugs.

FICI = FIC_Carvone_+ FIC_COL_ = (MIC_Carvone in combination_ / MIC_Carvone alone_) + (MIC_COL in combination_ / MIC_COL alone_).

FICI ≤ 0.5 indicates a synergistic effect, 0.5 ˂ FICI ≤ 4 indicates no interaction, and FICI > 4 indicates an antagonistic effect.

**Table S6** The MICs of carvone against experimental strains in different culture media

| **Strains** | **The MICs of carvone (μg/mL)** | |
| --- | --- | --- |
|  | **CAMHB^a^** | **LB broth^b^** |
| TL7733 | >512 | >512 |
| TL1671 | >512 | >512 |
| TL2917 | >512 | >512 |
| TL7929 | >512 | >512 |
| TL2314 | >512 | >512 |
| TL8126 | >512 | >512 |
| TL7333 | >512 | >512 |
| TL7508 | >512 | >512 |
| TL7505 | >512 | >512 |
| TL7548 | >512 | >512 |
| TL7440 | >512 | >512 |
| TL8269 | >512 | >512 |

^a^CAMHB, Cation-adjusted Mueller-Hinton broth.

^b^LB broth, Luria-Bertani broth.

**Table S7** Sequence of qPCR primers.

| **Gene name** | **Forward primer sequence (5’ to 3’)** | **Reverse primer sequence (5’ to 3’)** | | **Reference** | | |
| --- | --- | --- | --- | --- | --- | --- |
| *pmrA* | AATACTGCTGGCCGAGGAC | | GTCACCCATTCCACGGTATC | | (1) |  |
| *pmrB* | CGATCTTCACCCGCTTCTAC | | AAGTGCAGTTCGACGATGC | | (1) |  |
| *phoP* | TGCGCCACCACCTCTATAC | | GTATTCGCTGACCCGGTAGA | | (1) |  |
| *phoQ* | CCTGCTGGAGAACGCCTAT | | GACCCACAGCTCCGAGTAAC | | (1) |  |
| *mexX* | TGCGAAGAAGCAGCGGA | | CAGGCGACGGGTGACG | | (2) |  |
| *mexY* | CTACAACATCCCCTATGACACCT | | CATCACGGCGAACACCAG | | (2) |  |
| *lasI* | CGCACATCTGGGAACTCA | | CGGCACGGATCATCATCT | | (3) |  |
| *lasR* | CTGTGGATGCTCAAGGACTAC | | AACTGGTCTTGCCGATGG | | (3) |  |
| *pqsA* | GACCGGCTGTATTCGATTC | | GCTGAACCAGGGAAAGAAC | | (3) |  |
| *pqsR* | CTGATCTGCCGGTAATTGG | | ATCGACGAGGAACTGAAGA | | (3) |  |
| *rhlI* | GTAGCGGGTTTGCGGATG | | CGGCATCAGGTCTTCATCG | | (3) |  |
| *rhlR* | GCCAGCGTCTTGTTCGG | | CGGTCTGCCTGAGCCATC | | (3) |  |
| *16SrRNA* | AAGCAACGCGAAGAACCTTA | | CACCGGCAGTCTCCTTAGAG | | (1) |  |

**
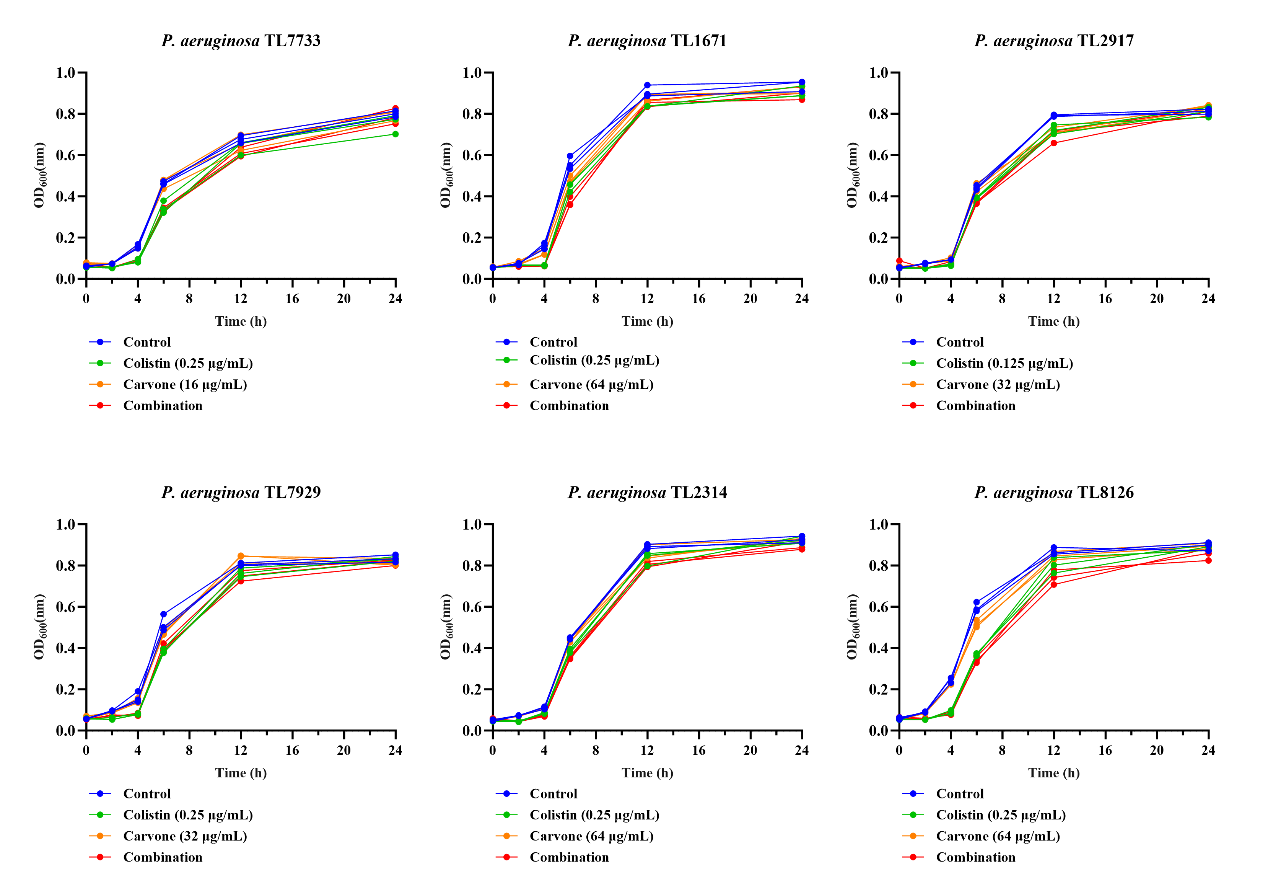
**

**Figure S2** The growth curve. The vertical axis represents the optical density values at 600 nm, with higher values indicating a greater number of bacteria and thus reflecting the growth status of the bacterial strain. The drug concentrations tested correspond to the sub-inhibitory concentration used in the crystal violet staining assay.

**The detailed** **procedure of some methods**

*1.Determination of Drug Sensitivity*

We employed the broth microdilution method to determine the minimum inhibitory concentrations (MICs) of the experimental strains. In accordance with the latest standards and guidelines published by the Clinical and Laboratory Standards Institute (CLSI), we utilized cation-adjusted Mueller-Hinton broth (CAMHB) for antimicrobial susceptibility testing of *P. aeruginosa*. The procedure was as follows: experimental strains were inoculated on blood agar plates and incubated at 37°C for 18-24 h to obtain fresh cultures. CAMHB was added to a 96-well microtiter plate at 100 μL per well. Subsequently, antibacterial agents were prepared at a concentration of 2048 μg/mL in CAMHB and added to column 12 of the 96-well plate using a pipette. A volume of 100 μL was added to each well and thoroughly mixed. Serial twofold dilutions were performed from column 12 to column 1, discarding 100 μL from the first column after mixing. This resulted in a 96-well plate with increasing drug concentrations from column 1 to column 12 (0.5-1024μg/mL). Fresh bacterial colonies were selected from the blood agar plates and adjusted to a turbidity equivalent to 0.5 McFarland standard (approximately 1.5 × 10^8^ CFU/mL) in sterile saline. The suspension was then diluted 1:100 in CAMH broth to achieve a final concentration of approximately 1.5 × 10^6^ CFU/mL. Using a pipette, 100 μL of this diluted bacterial suspension was added to each well containing the antimicrobial solution, resulting in a total volume of 200 μL per well. Consequently, the final concentration of antimicrobial agents in each well was half the initial dilution concentration (0.25-512μg/mL). Finally, the 96-well plates were incubated at 37°C for 16-20 h. The MIC was defined as the lowest antimicrobial concentration at which no visible bacterial growth, as evidenced by broth clarity, was observed. Drug susceptibility was evaluated using 2024 CLSI guideline breakpoints, with strains resistant to three or more antimicrobial categories designated as multi-drug resistance strains.

*2.* *The growth curve*

We verified that the sub-inhibitory concentrations of drugs used in the crystal violet staining assay do not affect bacterial growth by conducting the growth curve experiment. The experimental steps were similar to previous studies (4, 5). First, the bacterial strain was inoculated into Luria-Bertani (LB) broth containing different drugs, resulting in the blank control group, the colistin monotherapy group, the carvone monotherapy group, and the combination therapy group. The initial concentration of the bacterial strain in the final system was approximately 1.5 × 10⁶ CFU/mL, and the drug concentrations corresponded to those used in the crystal violet staining assay. The inoculated culture was placed in a constant temperature shaker at 37°C and 200 rpm for shaking incubation. At six time points (0, 2, 4, 6, 12, and 24 h), a certain volume of LB culture was taken from each medium and placed in a 96-well plate (200 µL per well) to measure absorbance at a wavelength of 600 nm. The growth curves were plotted with time on the horizontal axis (X-axis) and the optical density value at 600 nm on the vertical axis (Y-axis).

*3. Evaluation of in vivo safety*

In this experiment, healthy male CD - 1 (ICR) mice aged 28 - 34 days were used. The mice weighed 24 - 26 g. There were 3 mice in each group, with a total of 12 mice. The National Standard for Laboratory Animals of China (GB 14925-2010) was followed for housing the mice. Every experimental method was carried out in accordance with the Wenzhou Guidelines for Laboratory Animal Welfare and Ethics and approved by the Ethics Committee of Wenzhou Medical University's First Affiliated Hospital (Approval No: SYXK 2021-0017).

Referring to the mouse thigh infection model, mice were treated with a single - dose via intraperitoneal injection and divided into 4 groups in total: the PBS group, the COL monotherapy group (5 mg/kg/24 h), the carvone monotherapy group (20 mg/kg/24 h), and the combination group. Seven days later, the blood of the mice was collected by orbital blood sampling. Whole blood was used for blood routine counts. A portion of the blood was allowed to stand at room temperature for 1 h, then centrifuged at 3000 rpm/min at 4°C for 15 min. The supernatant was collected as serum for the detection of biochemical indices including liver function test indicators (ALT, alanine aminotransferase; AST, aspartate aminotransferase) and renal function test indicators (BUN, blood urea nitrogen; CREA, creatinine). Automated hematology and biochemical analyzers (Mindray BC-2800vet, Chemray 240, Chemray 420, and Chemray 800) facilitated blood analysis. Then, the mice were sacrificed by cervical dislocation. Their major organs (heart, liver, spleen, lung, and kidney) were preserved in a 4% formaldehyde solution. The tissues were then embedded in paraffin, yielding 5 µm sections. HE dye was employed for staining, and microscopy facilitated observation.

**References:**

1. Mozaheb N, Rasouli P, Kaur M, Van Der Smissen P, Larrouy-Maumus G, Mingeot-Leclercq M-P. 2023. A Mildly Acidic Environment Alters Pseudomonas aeruginosa Virulence and Causes Remodeling of the Bacterial Surface. Microbiol Spectr 11:e0483222.

2. Fan Z, Pan X, Wang D, Chen R, Fu T, Yang B, Jin Y, Bai F, Cheng Z, Wu W. 2021. Pseudomonas aeruginosa Polynucleotide Phosphorylase Controls Tolerance to Aminoglycoside Antibiotics by Regulating the MexXY Multidrug Efflux Pump. Antimicrob Agents Chemother 65.

3. Fekry M, Yahya G, Osman A, Al-Rabia MW, Mostafa I, Abbas HA. 2022. GC-MS Analysis and Microbiological Evaluation of Caraway Essential Oil as a Virulence Attenuating Agent against Pseudomonas aeruginosa. Molecules 27.

4. Andersen JB, Rybtke M, Tolker-Nielsen T. 2024. The dynamics of biofilm development and dispersal should be taken into account when quantifying biofilm via the crystal violet microtiter plate assay. Biofilm 8:100207.

5. Pan J, Zhang J, Hu P, Yao Z, Zhang X, Zhou T, Shen M. 2025. Daidzein-Decorated Gold Nanoparticles as a Novel Antimicrobial Strategy Against Carbapenem-Resistant Enterobacteriaceae. Int J Nanomedicine 20:7811–7827.
